# The burden of stroke in Africa: a glance at the present and a glimpse into the future

**DOI:** 10.5830/CVJA-2015-038

**Published:** 2015

**Authors:** Mayowa O Owolabi, Oyedunni Arulogun, Sylvia Melikam, Abiodun M Adeoye, Sally Akarolo-Anthony, Rufus Akinyemi, Donna Arnett, Hemant Tiwari, Mulugeta Gebregziabher, Carolyn Jenkins, Daniel Lackland, Bruce Ovbiagele, Albert Akpalu, Kwamena Sagoe, Fred Stephen Sarfo, Reginald Obiako, Lukman Owolabi

**Affiliations:** College of Medicine, University of Ibadan and University College Hospital, Ibadan, Nigeria; College of Medicine, University of Ibadan and University College Hospital, Ibadan, Nigeria; College of Medicine, University of Ibadan and University College Hospital, Ibadan, Nigeria; College of Medicine, University of Ibadan and University College Hospital, Ibadan, Nigeria; Harvard University, USA; Federal Medical Centre, Abeokuta, Nigeria; University of Alabama at Birmingham, USA; University of Alabama at Birmingham, USA; Medical University of South Carolina, USA; Medical University of South Carolina, USA; Medical University of South Carolina, USA; Medical University of South Carolina, USA; University of Ghana, Ghana; University of Ghana, Ghana; Kwame Nkrumah University of Science and Technology, Kumasi, Ghana; Ahmadu Bello University, Zaria, Nigeria; Bayero University, Kano, Nigeria

**Keywords:** stroke, Africa, epidemiology, incidence, mortality, prevalence

## Abstract

**Objective:**

Information on the current burden of stroke in Africa is limited. The aim of this review was to comprehensively examine the current and projected burden of stroke in Africa.

**Methods:**

We systematically reviewed the available literature (PubMed and AJOL) from January 1960 and June 2014 on stroke in Africa. Percentage change in age-adjusted stroke incidence, mortality and disability-adjusted life years (DALYs) for African countries between 1990 and 2010 were calculated from the Global Burden of Diseases (GBD) model-derived figures.

**Results:**

Community-based studies revealed an age-standardised annual stroke incidence rate of up to 316 per 100 000 population, and age-standardised prevalence rates of up to 981 per 100 000. Model-based estimates showed significant mean increases in age-standardised stroke incidence. The peculiar factors responsible for the substantial disparities in incidence velocity, ischaemic stroke proportion, mean age and case fatality compared to high-income countries remain unknown.

**Conclusions:**

While the available study data and evidence are limited, the burden of stroke in Africa appears to be increasing.

## Abstract

African countries are undergoing an epidemiological transition driven by socio-demographic and lifestyle changes.^1^ The burden of non-communicable diseases (NCD), including cardiovascular risk factors is increasing.^1^-^3^ Consequently, the incidence of stroke, a cardinal complication of cardiovascular risk factors, appears to be rising in Africa and other low- and middle-income country (LMIC) settings.^3^ Therefore, 86% of all stroke deaths around the world is contributed by LMIC in Africa and other continents.^4^ By contrast, the incidence of stroke appears to be declining in high-income countries.^4^,^5^

Ironically, there is insufficient information on the current epidemiology of stroke in African countries and other LMICs, where this knowledge is needed most. This is due to and contributes to deficient manpower and other resources to combat the epidemic.^6^ Accurate, up-to-date information on stroke burden is necessary for the development and evaluation of effective and efficient preventative acute care and rehabilitation programmes for stroke patients.

In an attempt to fill this gap, in 2013 and 2014, the Global Burden of Diseases (GBD) collaborators published data on the burden of stroke and stroke subtype based on multi-state models implemented in the software program DisMod III. These models were used to estimate the incidence, prevalence and disability-adjusted life years (DALYs) of ischaemic and haemorrhagic stroke in various countries across the globe but without specific focus on Africa.^2^,^7^

However, one meta-analysis^8^ focused solely on the prevalence and incidence of stroke in Africa, albeit with pooled data of uneven quality. In addition, there are some publications derived from primarily hospital-based data on the burden of stroke in Africa. Nevertheless, no recent publication has examined the burden of stroke in Africa, longitudinally and in its entirety,^9^ while identifying gaps in data and proposing appropriate interventions. This complete longitudinal picture is urgently needed for the design of appropriate interventions and the formulation of policy objectives within the framework of T4 (system and policy level) and T5 (global) translational science.^10^

The objectives of this systematic review were to examine the current burden and recent epidemiological trends of stroke in Africa using available resources (existing epidemiological data and models) while identifying knowledge gaps; and estimating the future burden and proposing a responsive and holistic action plan to control the epidemic. This comprehensive analysis will include data on incidence, mortality, case fatality, prevalence, DALYs, quality of life, vascular cognitive impairment, and cost of care.

## Methods

A systematic review of the literature was conducted according to the Preferred Reporting Items for Systematic Reviews and Meta-Analyses (PRISMA) guidelines.^11^ PubMed database was searched for ‘Africa’ combined with each of the following keywords: ‘stroke’, ‘cerebrovascular accident’, ‘intracerebral hemorrhage’ and ‘subarachnoid hemorrhage’. Further search was conducted using combinations of the keywords and sub-Saharan African countries such as ‘stroke Nigeria’. Other words were also used in association with the keywords, country names and Africa. These were ‘epidemiology’, ‘prevalence’, ‘incidence’ and ‘mortality’. Background references and citations were identified and screened to obtain more articles. Articles were included in the quantitative synthesis if they had an abstract in English, were published between January 1960 and October 2014, and described the epidemiological burden or determinants of stroke in Africa whether it was original or not.

**Fig. 1.**
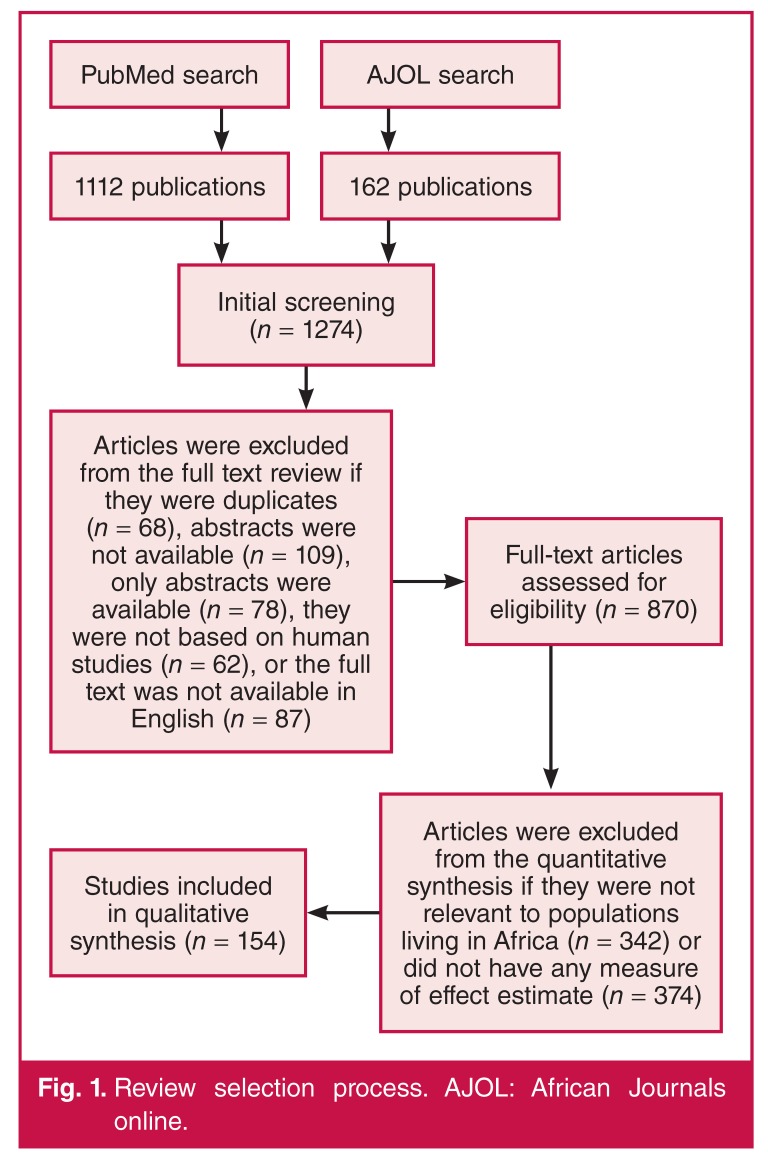
Review selection process. AJOL: African Journals online.

The search yielded a total of 1 274 articles (Fig. 1). All the articles were initially screened by one reviewer. We excluded 404 articles that were indexed in both PubMed and AJOL, did not have abstracts or full text in English, or were not based on human studies. Two reviewers read the remaining 870 articles in full to assess their eligibility for the quantitative synthesis. Fig. 1 shows the details of the review selection process. In addition, data were extracted from Global Burden of Diseases (GBD) model-derived figures.

Statistical analysis was performed to calculate percentage change in age-adjusted stroke incidence, mortality and DALYs for African countries between 1990 and 2010.

## Results and Discussion

## Incidence

Studies of stroke in Africa are mostly hospital-based case series. Hospital-based data cannot provide prevalence or incidence estimates (Tables 1, 2) because the population at risk (i.e. the denominator) is not known. Moreover, they are also affected by referral bias. Patients who die quickly from stroke or those with mild stroke may not be captured.^12^ Nevertheless, case series provide information about the relative frequency of stroke in comparison to other diseases requiring hospitalisation.

**Table 1:** Studies reporting crude incidence of stroke in Africa

			*Crude incidence per 100 000 per year*			
*Year*	*Country/location/setting*	*Author*	*Overall*	*Male*	*Female*	*Age*
	*Hospital-based*					
1984	Libya, Benghazi, urban	Ashok28	63	69	58	15+
1985	South Africa: Atteridgeville and Mamelodi, suburban areas of Pretoria, urban	Rosman29	101	108	93	20+
1991	Zimbabwe, Harare, urban	Matenga18	31	30	32	All
1993	Libya, Benghazi, urban	El Zunni30	48	52	42	15+
2006	Mozambique, Maputo, urban	Damasceno19	149	174	128	15+
	*Population/community-based*					
1975	Nigeria, Ibadan, urban	Osuntokun20	26	25	13	All
1993	Egypt, Sohag, mixed*	Kandil31	180	100	85	All
1993	Egypt, Sohag, urban	Kandil31	150	90	53	All
1993	Egypt, Sohag, rural	Kandil31	210	97	119	All
2006	Tanzania, Hai, rural	Walker32	95	107	77	All
2006	Tanzania, Dares Salaam, urban	Walker32	108	115	100	All
2007	Nigeria, Lagos, urban	Danesi24	25	28	21	All
2007	Egypt, Al-Kharga, mixed*	Farghaly22	250	270	230	All
2007	Egypt, Al-Kharga, rural	Farghaly22	230	250	220	All
2007	Egypt, Al-Kharga, urban	Farghaly22	260	280	240	All
2012	Egypt,Al Quseir, urban	El Tallawy21	181	212	150	20+

*Combined rates including both rural and urban communities.

**Table 2:** Population/community-based studies reporting prevalence of stroke survivors in Africa

			*Crude prevalence per 100 000*			
*Year*	*Country/location/setting*	*Author*	*Overall*	*Male*	*Female*	*Age*
1982	Nigeria, Igbo-Ora, rural	Osuntokun37	58	–	–	All
1985	Tunisia Kelibia, mixed*	Atia- Romdhane41	42	–	–	All
1988	Ethiopia, central Ethiopia, rural	Tekle Haimanot34	15	–	–	20–85
1993	Egypt, Sohag, mixed*	Kandil31	508	520	490	All
1993	Egypt, Sohag, urban	Kandil31	410	460	470	All
1993	Egypt, Sohag, rural	Kandil31	540	510	570	All
1994	Tanzania, Hai, rural	Walker42	127	155	103	15+
2002	South Africa: Agincourt Health and Population Unit, Limpopo province, rural	Connor43	243	188	296	15+
2006	Nigeria, Lagos, urban	Danesi38	114	151	69	All
2009	Benin, Cotonou, urban	Cossi44	460	610	360	15+
2009	Egypt, Al-Kharga, mixed*	Farghaly22	560	610	510	All
2009	Egypt, Al-Kharga, urban	Farghaly22	580	620	530	All
2009	Egypt, Al-Kharga, rural	Farghaly22	520	580	458	All
2010	Tanzania, Hai district, rural	Dewhurst39	2300	2971	1752	70+
2010	Egypt, Assuit, urban	Khedr35	963	1174	736	All
2013	Egypt, Qena, mixed*	Khedr40	922	1103	726	All

*Combined rates including both rural and urban communities.

Stroke is the leading cause of medical coma in Nigeria.^13^ It is also the leading cause of admissions from hypertension-related complications, accounting for 40% of hypertensive complications in the University of Port Harcourt Teaching Hospital, Nigeria.^14^ In several studies from the West African sub-region, it emerged as the leading cause of adult neurological admissions, constituting up to 65% of such admissions.^15^

Furthermore, a steady increase in stroke admissions has been observed in some institutions that have monitored their stroke admissions over time. In Tanzania, stroke admissions increased from 23 per 100 000 in 1935 to 86 per 100 000 in 1962.^16^ In Ghana, the number of stroke patients admitted per year increased from about 50 in 1960 to 622 in 1993, and the percentage of total adult medical admissions due to stroke increased from less than 2% in 1960 to about 12% in 1993.^16^

Stroke admissions to hospital are clearly rising in Africa. Although this could be due to increased patronage of orthodox medicine, increasing stroke incidence in an ageing population in the throes of epidemiological transition is a more plausible explanation.^12^

Using hospital data, five studies estimated stroke crude incidence rates ranging from 31/100 000 per year in Harare, Zimbabwe in 199^18^,^17^,^18^ to 149/100 000 per year in Maputo, Mozambique in 2006^8^,^17^,^19^ (Table 1). In a meta-analysis by Adeloye, the pooled estimate of 77.39/100 000 per year (95% CI = 51.31–103.48) from hospital-based studies^8^ was lower than from community-based studies. This may suggest that the available hospital-based African studies underestimated stroke incidence as a result of exclusion of fatal or mild cases who do not present in these hospitals.

Stroke incidence, estimated on the basis of representative community samples with rigorous case ascertainment and accurate diagnosis over a minimum period of three years, provides far more information about stroke burden than hospital-based studies. Nevertheless, such studies require considerable resources and rigorous methods.^16^

There are several community-based incidence studies from sub-Saharan Africa (Table 1). From the (1973–75) stroke registry in Ibadan, Nigeria, the crude annual incidence of first-ever stroke was 26 per 100 000. However, this is likely an underestimate, because of difficulties with case ascertainment resulting from the very large population, small study staff, and non-inclusion of those who patronised traditional healers.^20^

In Tanzania, stroke incidence was recorded in two demographic surveillance sites: Hai (rural) and Dar-es-Salaam (urban) from 2003–2006. Patients with stroke were identified by the use of a system of community-based investigators and liaison with local hospital and medical centre staff. Patients who died from stroke before recruitment were identified via verbal autopsy, which might have included non-incident strokes.^16^ Overall crude annual stroke incidence rates were 94.5 per 100 000 in Hai and 107.9 per 100 000 in Dar-es-Salaam (Table 1). When age-standardised to the WHO world population, annual stroke incidence rates were 108.6 per 100 000 in Hai and 315.9 per 100 000 in Dar-es-Salaam.^16^

Age-standardised stroke incidence rates in Hai were similar to those reported in developed countries. However, age-standardised incidence rates in Dar-es-Salaam were higher than those published from developed countries. This could be because of differences in the prevalence of risk factors, which emphasises the importance of health screening at a community level.^16^

A recent door-to-door survey of every household in Al Quseir (urban), Egypt^8^,^17^,^21^ from 2009 to 2012 reported a crude annual incidence of 181 per 100 000 population but the age-standardised incidence was not calculated (Table 1). Furthermore, Farghaly *et al.* performed a door-to-door screening in Al Kharga district, Egypt,^8^,^17^,^22^ from 2005 to 2009 and reported a crude annual incidence of 250 per 100 000 population (Table 1). Although the age-standardised incidence was likely to be higher than that in Tanzania (Dar-es-Salaam), which is the global highest,23 it was not reported.

Generally, population-based crude incidence rates were higher than hospital-based rates, ranging from 26.0/100 000 person years in Ibadan, Nigeria in 1979,^8^,^17^,^20^,^24^ to 250/100 000 person years in Al-Kharga, Egypt in 2007^8^,^17^,^22^ (Table 1). The random-effects meta-analysis of crude population-based incidence rates was 112.94/100 000 person years (95% CI = 90.7–135.0).8 However, this meta-analysis included incidence studies with incomplete case ascertainment,^24^ conducted over one year rather than the recommended three-year period.^8^,^12^,^16^,^17^ The studies reporting low rates, therefore, could have been marked by underestimation of the stroke burden in Africa, and the pooled estimate8 reported might therefore be much lower than the true rates.

Crude rates provide valuable information that reflects the public health burden of stroke, given the age distribution for the country (i.e. if a specific country has a large number of strokes because it has a relatively large elderly population, they must nevertheless care for this larger number of people), whereas adjusted rates allow a more comparable basis between the risk of stroke across the life course of residents of the country and for comparison between countries.^23^ Crude rates underestimate the impact of stroke on a country, particularly when strokes are occurring at younger ages, as occurs in Africa.

Nevertheless, the annual crude incidence rate in Egypt was higher than reports by Béjot *et al.* in France (113.5 per 100 000), Corso *et al.* in Italy (223 per 100 000), Vega *et al.* in Spain (113.5 per 100 000),^22^ and Pandian *et al.* in India (119 to 145/100 000).^25^,^26^ The age-standardised incidence of stroke in Tanzania was similar to the rates in China where the age-standardised incidence of first-ever stroke per 100 000 person years increased rapidly from 124.5 in 1992–1998 to 190.0 in 1999–2005, and to 318.2 in 2006–2012.^27^

**Fig. 2.**
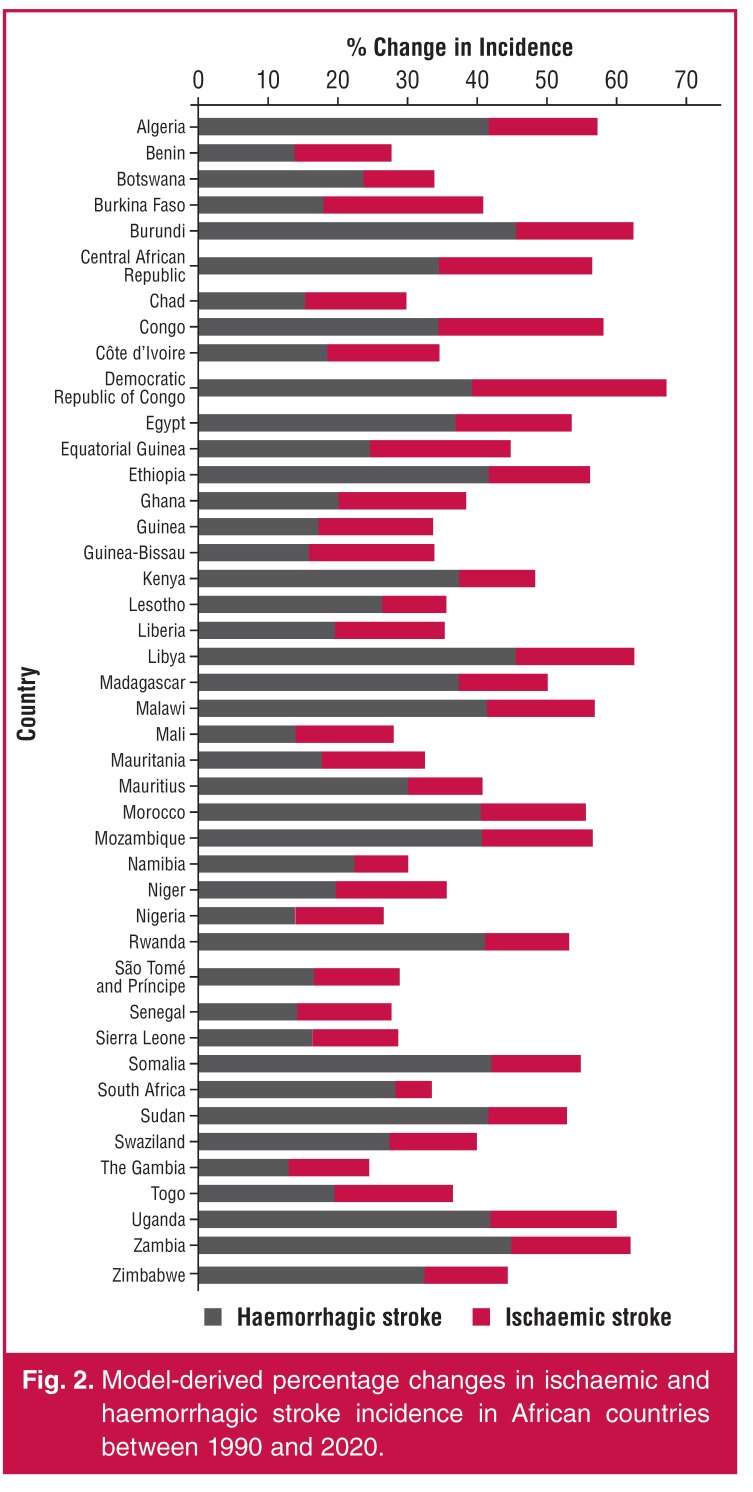
Model-derived percentage changes in ischaemic and haemorrhagic stroke incidence in African countries between 1990 and 2020.

Unfortunately, no rigorously conducted stroke-incidence study has been performed twice in the same location to provide secular trend data on the incidence ‘velocity’ (trend) of stroke in Africa. Using the GBD data (Fig. 2), increase in age-standardised ischaemic stroke incidence from 1990 to 2010 ranged between 5.2% (South Africa) and 27.8% (DRC, Table 3).

**Table 3:** Estimates of average percentage change over 1990 to 2010 in age-adjusted incidence, mortality and DA LYs of stroke in Africa

*Rates per 100 000 person years*	*1990 (mean, SD)*	*2010 (mean, SD)*	Min. change* (%)	*Country with min. change*	Max. change* (%)	*Country with max. change*	Mean/ median change** (%)	*SD*	*p-value*
Age-standardised incidence ischaemic	129.4, 15.1	148.4, 16.3	5.18	South Africa	27.8	Democratic Republic of Congo	+14.8	4.05	< 0.001
Age-standardised incidence haemorrhagic	58.9, 11.0	75.2,12.9	13.0	The Gambia	45.7	Burundi	+28.7	11.1	< 0.001
Age-standardised mortality ischaemic	53.3, 15.2	48.1, 12.5	–45.5	Mauritius	95.0	Burkina Faso	–7.5**		0.001
Age-standardised mortality haemorrhagic	69.2, 20.1	58.8, 16.9	–52.2	Equatorial Guinea	67.9	Burkina Faso	–12.7**		< 0.001
DALYs lost ischaemic	853.8, 231.7	756.1, 192.7	–53.1	Mauritius	79.0	Burkina Faso	–10.3**		< 0.001
DALYs lost haemorrhagic	1574.7, 451.1	1287.1, 383.9	–57.4	Equatorial Guinea	51.6	Zimbabwe	–18.9**		< 0.01

*Countries with the minimum and maximum changes in rates are depicted. **Median percentage change.

Overall, in Africa, there was significant (*p* < 0.001) mean increase in age-standardised ischaemic stroke incidence of 14.8% (± 4.1%) between 1990 and 2010. Similarly (Fig. 2), increase in age-standardised haemorrhagic stroke incidence from 1990 to 2010 ranged between 13.0% (the Gambia) and 45.7% (Burundi, Table 3). Overall, in Africa, there was significant (*p* < 0.001) mean increase in age-standardised haemorrhagic stroke incidence of 28.7% (± 11.1%) between 1990 and 2010. Therefore, the incidence of stroke in Africa is not only among the highest in the world, the incidence velocity is also very high.

Urbanisation and other socio-demographic and lifestyle changes in Africa, as in other parts of the developing world, are increasing rapidly, and the results from this study suggest that, in the absence of effective preventive measures, this is likely to lead to further substantial increases in stroke incidence.

## Prevalence

A retrospective chart review of clinically and CT-diagnosed stroke patients evaluated between January 2000 and March 2005 in Tikur Anbessa tertiary referral and teaching hospital (Addis Ababa, Ethiopia) showed that stroke accounted for 5% of all head CT indications done in Ethiopia.^33^ A prevalence rate could not be calculated in the absence of the number in the referral base.

Community-based studies constitute the best way to determine the true prevalence of stroke, although they are very rare in Africa due to lack of manpower and research funds. Estimating the prevalence of stroke survivors in the community is complicated by the difficulty in making a retrospective and yet accurate diagnosis of stroke and stroke type months or years after the event.^16^ Estimations are also biased by underrepresentation of fatal cases.^16^ Therefore, prevalence, which depends on incidence and case fatality, is better estimated from incidence studies of first-ever stroke and survival. However in sub-Saharan Africa, incidence studies are very rare and difficult to conduct.^16^

There were many population-/community-based studies reporting crude prevalence of stroke survivors with prevalence rates ranging from 15/100 000 population in Ethiopia in 1988,^8^,^17^,^34^ to 963/100 000 population in Egypt in 2010 (Table 2).^8^,^17^,^34^,^35^ The low prevalence rate recorded in Ethiopia in 1988, included in the meta-analysis, may have been due to the high fatality rates from stroke, which have generally been reported in many parts of Africa.^8^,^17^,^34^,^36^ It may also reflect low stroke incidence in rural Ethiopia at that period, or simply that patients with mild strokes who had recovered were not detected. Moreover, the Ethiopian study was a broad door-to-door survey of neurological disorders in the community, which could imply that active case recognition of specific stroke cases may be less rigorous.^8^

In 1982, in Igbo Ora, Nigeria, stroke had an estimated crude prevalence of 58 per 100 000 (Table 2). However, the denominator population was far too small to establish stroke prevalence accurately.^37^ In 2005 to 2006, another study conducted in Lagos, Nigeria yielded a crude prevalence rate of stroke of 114/100 000 persons.^38^ This may suggest at least a doubling of the stroke prevalence in Nigeria. As reported by several other studies, males were more affected (males:female = 1.51) and age was a strong risk factor with prevalence of nearly 5% for those in the ninth decade of life.^38^

Stroke-prevalence studies in demographic surveillance sites that provide an accurate denominator have arguably provided the most accurate measures of stroke burden in recent years, despite their limitations.^16^ The largest study of the prevalence of disabling hemiplegic stroke in sub-Saharan Africa was done in 1994 in the rural Hai district of Tanzania (Table 2).^16^ It provided an age-standardised (Segi world population) prevalence of disabling stroke of 154 per 100 000 in men and 114 per 100 000 in women over 15 years of age.

In 2001, a stroke-prevalence study in Agincourt, rural South Africa, with diagnosis of stroke based on the WHO definition of stroke, provided an age-standardised (Segi world population) stroke prevalence of 290 per 100 000 people over the age of 15 years (male: 281 per 100 000, females 315 per 100 000).^16^

The rural Tanzanian (1994) and Agincourt studies (2001) both have the advantage of accurate denominators and careful assessment of people who screened positive for stroke. However, the higher prevalence of stroke in Agincourt may be because Agincourt is further along the epidemiological transition, or due to the fact that the Tanzanian study included only disabling hemiplegic stroke.^16^ A repeat rural Tanzanian study^8^,^17^,^39^ showed an increase in prevalence per 100 000 population from 127 among people aged 15 years and above in 1994 to 2 300 in 2010 among people aged 70 years and above (Table 2).^8^,^16^,^17^,^39^ Similarly, as shown in Table 2, comparison between studies performed in Egypt in 1993 and 2009 showed an increase in prevalence per 100 000 population from 508 to 560 (mixed) and 410 to 580 (urban).

Supporting this increase, two recent studies in Egypt produced crude prevalence rates of 922^40^ and 963 per 100 000 population (Table 2), with an age-adjusted local prevalence rate of 699.2/100 000 and an age-adjusted prevalence relative to the standard world population of 980.9/100 000.^35^ There was a significantly higher prevalence of ischaemic (895/100 000) than haemorrhagic (68/100 000) stroke. Stroke prevalence was the same in rural and urban areas but significantly higher in illiterate (2 413/100 000) than literate participants (3 57/100 000).^35^

Overall in Africa, the observed population-based prevalence rates of stroke survivors were generally high and rising, with a pooled crude prevalence rate of 387.9/100 000 population (which may be an under-estimate due to the inclusion of the Ethiopian study among the 11 studies used for the estimate^8^) and a range of up to 963 per 100 000 all population.^8^,^17^,^22^ This prevalence lies within the range of that recorded in other LMICs (500–1 000 per 100 000) and is in agreement with that found in India (550 per 100 000), but higher than that recorded in Saudi Arabia (180 per 100 000) and Italy (140 per 100 000).^22^ The high prevalence of stroke in the study population may reflect the increased exposure to risk factors for stroke due to ongoing epidemiological and demographic transitions.

## Mortality

Cause-of-death data from Africa are usually not from standard vital registration, but are predominantly gathered from verbal autopsy studies, police reports, sibling histories, and burial and mortuary reports. With the exception of a few higher-quality studies, most data on CVD in Africa are from small community surveys and hospital-based registries.^3^,^23^

Hospital-based data show that NCDs are the leading cause of death in Africa. In a rural hospital in Nigeria, NCDs constituted 63% of deaths, with stroke being the leading NCD cause.^45^,^46^ Similarly, hypertension-related NCD deaths led by stroke constituted the leading cause of death in a Tanzanian hospital from 2009 to 2011.^47^

Based on verbal autopsies from burial surveillance of 58 010 deaths in Addis Ababa from 2006 to 2009, about 11% of the deaths were attributed to stroke. The mortality rate increased with age (15–34 years: 1%; 35–54 years: 7%; 55–74 years: 16%; > 74 years: 18%) but there were no differences by gender.^48^

The Agincourt community-based study in South Africa found that stroke caused 6% of all deaths between 1992 and 1995.^16^ Stroke was the most common cause of death in the age group 55–74 years, and the second most common cause of death in the age group 35–54 years and the > 75 years group.^16^ The crude stroke mortality rate was 127 per 100 000 over age 35 years.^16^ In a verbal autopsy study in Tanzania, stroke caused 5.5% of adult deaths in three regions [Dar-es-Salaam (urban), Hai (prosperous rural) and Morogoro (impoverished rural)].^16^

Age-specific stroke mortality rates in Agincourt and the three regions of Tanzania mentioned above may be as high as in England and Wales, and perhaps higher in younger age groups, but larger studies based on accurate vital registration data are clearly needed.^16^ Such data will produce evidence of any change in stroke mortality rate particularly as lifestyle, cardiovascular risk burden, population age structure, relative stroke incidence and case fatality rates change in Africa.

The GBD dealt with the problem of absent or low-quality epidemiological data from sub-Saharan Africa by incorporating covariates (CVD risk factors, national income, differences in measurement method) and ‘borrowing strength’ from nearby regions and years of observation in CODEm and DisMod-MR models; and using standard assumptions about the relationship between disease-specific incidence, prevalence, case fatality, and mortality in DisMod-MR models.^3^ The ensemble approach combined different model results developed with different combinations of covariates and statistical approaches.^2^,^7^,^49^

Using these models, the leading CVD cause of death and disability in 2010 in sub-Saharan Africa was stroke.^3^ Furthermore, Krishnamurthi^50^ reported higher age-adjusted stroke mortality rates for haemorrhagic stroke in sub-Saharan Africa than in North America and Europe.

Overall, the GBD generated an age-standardised stroke mortality rate of between 52.0 and 136.7 per 100 000 people for 2010.^2^,^49^ Indeed there was as much as a 10-fold difference between the lowest stroke mortality rates, seen primarily in developed nations, and the highest mortality rates, seen primarily in numerous countries across central and western Africa and other LMIC.^5^

In addition to comparing the mortality rates at a given time point, it is also important to examine the trend to forecast future disease burden. In the Seychelles, mortality rates (per 100 000, age-standardised to WHO standard population) decreased from 250/140 (male/female) to 141/86 for stroke, corresponding to 44/39% over 22 years. However, overall stroke mortality rates remained high, emphasising the need to strengthen neurological disease prevention and control.^2^,^49^

**Fig. 3.**
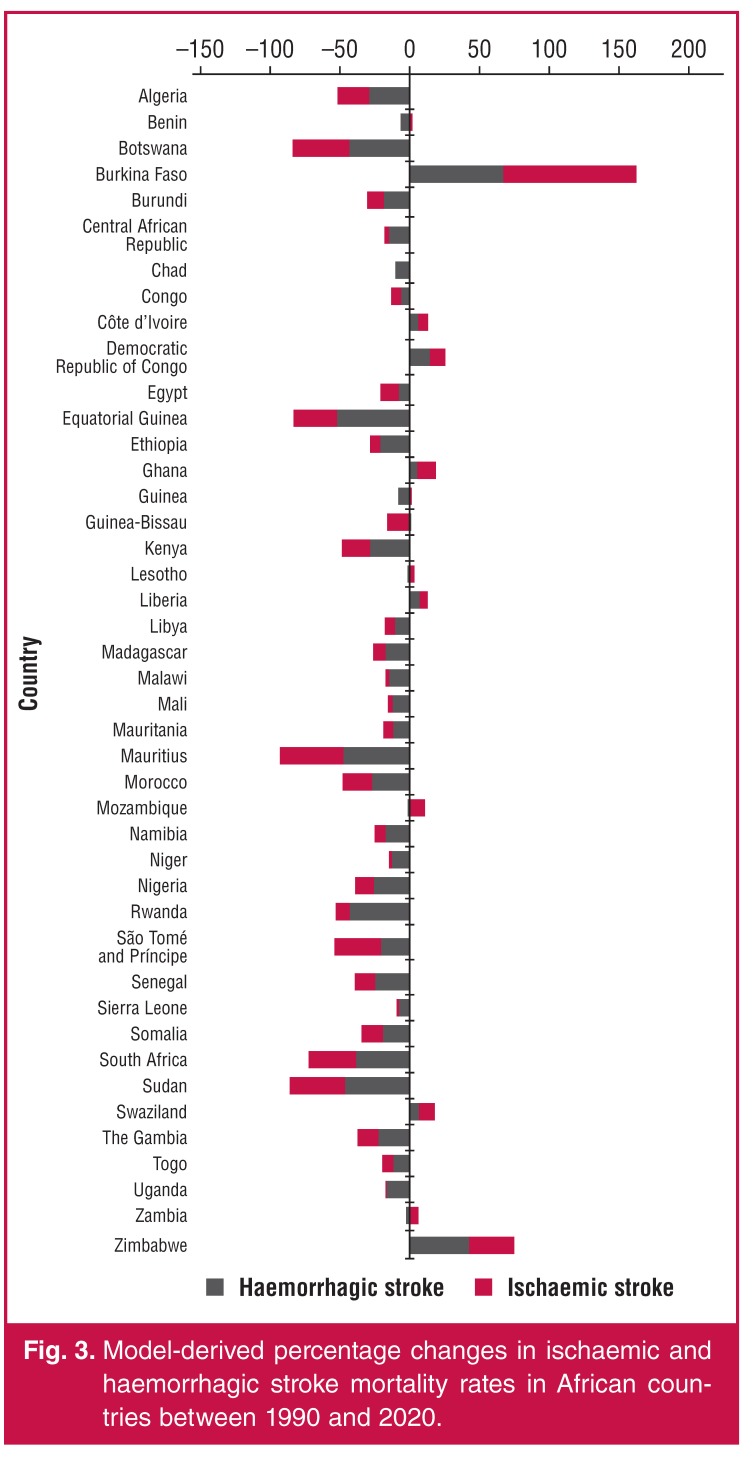
Model-derived percentage changes in ischaemic and haemorrhagic stroke mortality rates in African countries between 1990 and 2020.

Using the GBD data (Table 3, Fig. 3), percentage change in age-standardised ischaemic stroke mortality rates from 1990 to 2010 ranged between –45.5% (Mauritius) and 95.0% (Burkina Faso). Overall, in Africa, there was a statistically significant (*p* = 0.001) median change in age-standardised ischaemic stroke mortality rates of –7.5% between 1990 and 2010. Similarly, (Table 3), change in age-standardised haemorrhagic stroke mortality rates for the same period ranged between –52.2% (Equatorial Guinea) and 67.9% (Burkina Faso). Overall, in Africa, there was significant (*p* < 0.001) median change in age-standardised haemorrhagic stroke mortality rates of –12.7% between 1990 and 2010.

In the GBD, although age-standardised mortality rates decreased between 1990 and 2010 in Africa, crude mortality rates increased in sub-Saharan Africa, south Asia, and central and Latin America, but decreased in high-income North America, western and central Europe, North Africa and the Middle East, Australasia, and high-income Asia Pacific.^2^,^49^ These changes are in keeping with the expected increase in crude mortality rate due to the increasing crude incidence.^23^

Africa is at an earlier stage of health transition with a higher ratio of stroke death to coronary death.^51^,^52^ As a population undergoes health transition, the pattern of vascular disease is thought to change from one dominated by stroke, with a high proportion caused by cerebral haemorrhage, to a pattern dominated by atherosclerotic stroke, coronary heart disease and peripheral vascular disease.^16^,^53^ This scenario is expected to occur in Africa, as suggested by a study exploring the relationship of vascular risk factors to stroke type among Africans, in which we found age above 61 years and previous transient ischaemic attack to be associated with ischaemic stroke, while uncontrolled hypertension predicted haemorrhagic stroke.^54^

With increasing proportion of the population over 61 years and improving control of blood pressure, the proportion of ischaemic stroke is expected to rise in African countries.^54^ Therefore, relevant components of the stroke-intervention quadrangle (described below) should be tailored toward this need to mitigate the burden.^54^

## Case fatality

Hospital-based studies have demonstrated a one-month case fatality rate of between 27 and 46% in Africans.^16^,^32^,^55^ In the hospital-based INTERSTROKE study, the one-month case fatality rate for stroke was 22% in the African region compared to 4% in high-income countries.^56^ Reports of post-stroke deaths in sub-Saharan Africa are, however, unreliable due to factors such as limited death certification and lack of coverage of primary healthcare services.^55^ Post-stroke case fatality rates should ideally be calculated using community-based studies because of the heterogeneity of stroke type and severity, and the likelihood that many patients are not admitted to hospital.^16^

In the Ibadan community-based stroke registry (1975), case fatality rate at three weeks was 35% for all strokes and highest for cerebral haemorrhage (61%) and subarachnoid hemorrhage (62%). However, this case fatality rate may not be very reliable because stroke types had most probably been diagnosed unreliably without CT scanning.^20^,^57^

In the Tanzanian community-based incident stroke study (2003), case fatality rate was 28.7% at 28 days and 84.3% at three years. The 28-day case fatality rate was at the lower end of rates reported for other LMIC, even when including those identified by verbal autopsy, while the three-year case fatality rates were notably higher than seen in most developed-world studies. Recent studies from the developed world suggest three-year case fatality rates of 43 to 54% and five-year case fatality rates of 53 to 60%.^32^

In a South African study (published in 2012), 25.5% of patients died within three months of discharge and 38% within the 12-month follow-up period.^58^ This high fatality rate may be due to the severe scarcity and prohibitive costs of facilities and human resources for investigations, acute care and rehabilitation of stroke patients in Africa.^6^ The region has the lowest neurologist-to-population and doctor-to-population ratio in the world,^6^ with an average of one neurologist to one million people in comparison to one to 100 000 in high-income countries.^6^

With high proportion of the population living below the poverty line, the few available facilities for investigation and care of stroke patients are not accessible to most of the population who have to pay out of their pockets.^6^,^59^ For instance, there is probably only one multidisciplinary holistic neuro-rehabilitation centre in East, West and Central Africa.^60^, ^61^

## Disability-adjusted life years

Direct studies of DALYs due to stroke are very rare in Africa. The burden of disease due to stroke in South Africa (2008) was 564 000 DALYs.^62^ Of this, 17% was contributed by years lost to disability (YLD) (14–20% in sensitivity analysis).^62^ The estimated DALYs lost due to stroke was 1 230 per 100 000 in Angola, Africa, compared to 200 per 100 000 in Switzerland, Europe in 2002.^53^,^63^

**Fig. 4.**
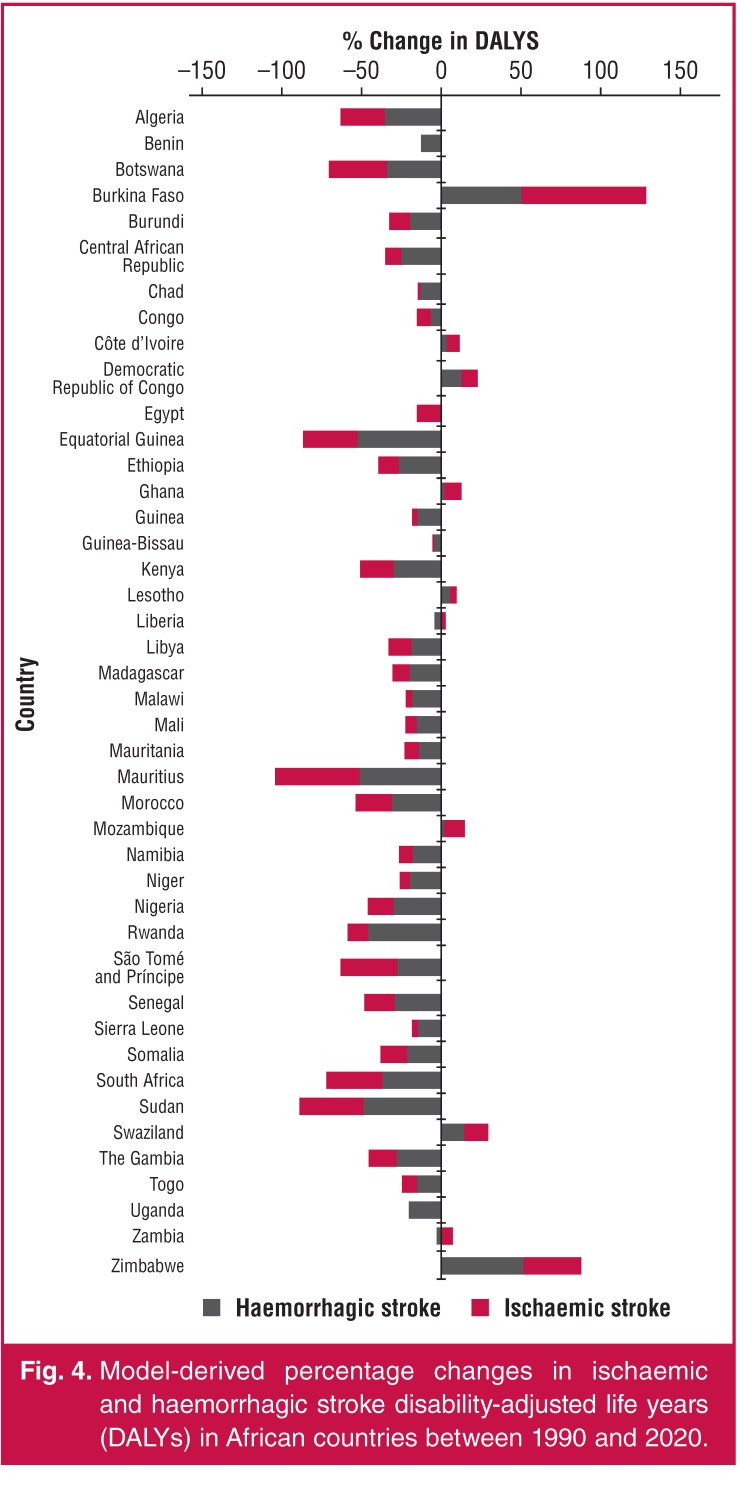
Model-derived percentage changes in ischaemic and haemorrhagic stroke disability-adjusted life years (DALYs) in African countries between 1990 and 2020.

Using the GBD data (Table 3, Fig. 4), percentage change in age-standardised ischaemic stroke DALYs from 1990 to 2010 ranged between –53.1 (Mauritius) and 79.0 (Burkina Faso). Overall, in Africa, there was significant (*p* < 0.001) median change in age-standardised ischaemic stroke DALYs of –10.31 between 1990 and 2010. Similarly (Table 3), change in age-standardised haemorrhagic stroke DALY for the same period ranged between –53.8 (Equatorial Guinea) and 51.6 (Zimbabwe).

Overall, in Africa, there was a statistically significant (*p* < 0.001) median change in age-standardised haemorrhagic stroke DALYs of –18.9 between 1990 and 2010. However, stroke remained the leading cause of cardiovascular DALYs in sub-Saharan Africa, increasing from 5 930 040 (39.5%) in 1990 to 7 824 920 (52.0%) of CVD DALYs in 2010.^3^

## Stroke type and risk factors

The proportion of haemorrhagic stroke in Africa ranges from 29 to 57%, in comparison with 16 to 20% in North America.^53^ In the INTERSTROKE study, haemorrhagic stroke was 34% in Africa and 9% in high-income countries.^56^ This suggests a higher burden of uncontrolled hypertension in Africa, because the proportion of haemorrhagic stroke in a population seems to correlate with the prevalence and severity of uncontrolled hypertension.^16^,^32^,^53^-^55^

Up to 98% of stroke patients in Africa have hypertension.^32^,^53^,^55^ Ischaemic stroke is more associated with diabetes mellitus, cardiac disease, age above 61 years and previous transient ischaemic attacks.^54^ The population-attributable ratio of stroke due to hypertension in South Africa in 2000 was 50%,^64^ and 60% in North Africa.^65^

## Hypertension

Hypertension, once rare in West Africa, is emerging as a serious endemic threat. It has been referred to as a silent killer, as it often has no early detectable symptoms despite being a major cause of serious health conditions, including heart disease, stroke and renal disease.^66^ Of the 10 predominant modifiable risk factors accounting for 90% of the risk of stroke, hypertension is the strongest.^56^

Prevalence rates for hypertension vary across and within regions in Africa. An analysis of all national data in Zimbabwe in the 1990s found that between 1990 and 1997, the national crude prevalence of hypertension increased from one to 4%. According to Adedoyin *et al.*, up to 36.6% of adult Nigerians were hypertensive in 2008.^67^

The impact of migration from rural to urban areas was demonstrated in a longitudinal study in Kenya, in which moving from a rural to an urban setting produced significant increases in blood pressure within a short time. Growing migration from rural to urban areas also portends a worsening prevalence of hypertension as migrants adopt lifestyle changes in physical activity, dietary habits and stress levels. Regardless of gender or type of community, advancing age is associated with an increased prevalence of hypertension, and this implies a greater burden of hypertension (and indeed stroke)^68^ as population aging occurs in Africa.^67^-^69^

Several surveys have demonstrated a very low prevalence of hypertension awareness and control (BP < 140/90 mmHg) in Africa. In Tanzania, slightly less than 20% of hypertensive subjects were aware of their diagnosis, approximately 10% of them were treated, and < 1% were controlled.^70^ A survey in Ghana showed that 34% were aware of their condition, of whom 18% were treated and only 4% were controlled. However, in the United States, 69% of hypertensive subjects were aware of their diagnosis, 58% of them were treated, and 31% were controlled.^70^ The low prevalence of awareness, treatment, and control of hypertension poses a serious challenge for stroke prevention in Africa.^70^ This scenario also applies to several other NCDs such as diabetes mellitus and dyslipidaemia, which are on the increase in Africa.^66^

## Type 2 diabetes mellitus

According to International Diabetes Federation (IDF), the current estimated prevalence rate of type 2 diabetes in Africa is about 2.8%. Countries such as Malawi and Ethiopia have rates under 2%, whereas Ghana, Sudan and South Africa have prevalence rates over 3%.^66^ Currently, there are 10.4 million individuals with diabetes in sub-Saharan Africa, representing 4.2% of the global population with diabetes. By 2025, it is estimated that this figure will have increased by 80% to reach 18.7 million in this region, with a higher prevalence in the urban areas.^66^ Studies indicate that an aging population, coupled with rapid urbanisation, is expected to lead to the increasing prevalence of diabetes in Africa.^66^

## Dyslipidaemia

Dyslipidaemia has emerged as an important risk factor in Africa. For example, Norman and colleagues found that high cholesterol levels (≥ 3.8 mmol/l) accounted for 59% of ischaemic heart disease and 29% of ischaemic stroke burden in adults aged 30 years and over.66 The prevalence of dyslipidaemia, especially cholesterol has been shown to vary across regions in Africa.

In a study of healthy workers in Nigeria, 5% of the study population had hypercholesterolaemia, 23% elevated total serum cholesterol levels, 51% elevated low-density lipoprotein (LDL) cholesterol levels and 60% low high-density lipoprotein (HDL) cholesterol levels, with females recording better overall lipid profiles.66 Population-based studies in Tanzania and Gambia also showed elevated total serum cholesterol levels of > 5.2 mmol/l in up to 25% of people aged > 35 years. Elevated cholesterol levels appear to be more prevalent in urban areas and among the higher socio-economic classes. 66

## Other factors

The epidemic of stroke, hypertension, diabetes and dyslipidaemia in Africa is driven by multiple factors working collectively. Obesity and lifestyle factors such as poor diet, sedentary lifestyle and smoking contribute to the increasing rates of stroke in Africa.

In a meta-analysis among West African populations, the prevalence of obesity was 10.0%. A study in Benin found that abdominal obesity was positively associated with increased probability of the metabolic syndrome. Obesity was a predominant risk factor for women compared to men, but smoking was mostly a risk factor for men.66 Additionally, structural and system-level issues such as lack of infrastructure for healthcare, urbanisation, poverty and lack of government programmes also drive this epidemic and hamper proper prevention, surveillance and treatment efforts.66

Carotid atherosclerosis measured by increased carotid intima–media thickness (CIMT) and carotid diameter have been associated with stroke among Africans.54,71-73 Furthermore white matter hyperintensities may be a risk factor for stroke in Africans.74

Elevated homocysteine levels (associated with cardiovascular endothelial injury)75,76 and the metabolic syndrome (implying concomitant hypertension, obesity, dyslipidaemia, and/or hyperglycaemia)77 have also been documented as risk factors for stroke in Africans.

## Unique aspects of stroke survivors in Africa

In Nigerian Africans, stroke impairs all facets of health-related quality of life (HRQOL), particularly domains in the physical sphere (physical, cognitive, psycho-emotional and eco-social domains). The severity of impairment correlates with stroke severity.^78^-^80^ Many of these disabling strokes occur in young people. Stroke occurs at a younger mean age of 57 years in Africa compared to 66.0 years in high-income countries (HICs); in those ≤ 45 years: 24% in Africa, 8% in HICs).^53^,^56^

Overall, stroke tended to occur in a younger population in Africans compared to high-income countries.^53^,^56^ This may be due to genetic factors, a high proportion of undiagnosed and uncontrolled hypertension, the shorter life expectancy in African countries and a higher proportion of younger people.^53^,^81^

Stroke is a leading cause of late-onset seizure disorder among Africans.^82^ It accounts for 22.5% of seizures after the age of 25 years.^82^ In a Nigerian study, the most common seizure type was simple partial, while the most common electro-encephalographic finding was the presence of focal epileptiform discharges, followed by focal slowing.^82^ At the three-month follow up, 52% of the patients had good seizure control.^82^ In other studies, 48.3% of Nigerian stroke patients had vascular cognitive impairment,^83^ while major depression was found among 30% of African stroke patients.^84^ Despite these deleterious consequences of stroke, there is poor community awareness of its risk factors and warning signs in Ghana,^84^,^85^ and poor awareness of its risk factors and features among hospital workers in Nigeria.^83^

## Cost of care

The economic burden of stroke is considerable. The cost of stroke for the year 2002 was estimated to be as high as $49.4 billion in the United States, while costs after hospital discharge were estimated to amount to 2.9 billion Euros in France.^16^,^70^,^86^,^87^ Clearly, even a fraction of such amounts can cause enormous economic damage to low-income countries.^70^

There are very few studies on the cost of stroke care in Africa. A study in Togo estimated direct cost per person of 936 Euros in only 17 days, about 170 times more than the average annual heath spend of a Togolese.^88^ Subsidising and improving post-stroke care may help to reduce stroke case fatality rates and morbidity in Africa.^89^

## The gaps

Although age-standardised rates of stroke mortality have decreased worldwide in the past two decades, the absolute number of people who have a stroke every year, stroke survivors, related deaths, and the overall global burden of stroke (DALYs lost) are great and increasing. Further studies are needed to improve understanding of stroke determinants and burden worldwide, and to establish causes of disparities, and changes in trends in stroke burden between countries of different income levels.^2^

In Africa, despite the enormous and growing burden, numerous gaps have been identified in the required data and interventions to tame the scourge. For most of the direct observational studies and models, a time lag of about three to six years was observed between data collection, analyses and publication. There is incomplete understanding of the pattern and determinants of stroke occurrence, type, subtype, outcome, complications and burden. Furthermore, the incidence, prevalence, relative risk, and population-attributable risks (PAR) of genomic and environmental risk factors for stroke among Africans are not known. Assessment of hypertension and its risk factors is needed.^90^ Accurate population demographic information essential for determining rates is also needed.

Moreover, causes of disparities and changes in trends of stroke burden in LMIC/HIC, whites/Africans, as well as the genomic architecture of stroke among Africans are unknown. The peculiar genomic, gene–environment and environmental risk, and protective factors for stroke occurrence, pattern, type, subtype, outcome and current incidence velocity among people of African ancestry is unclear. There is also a need for indicators and determinants of blood pressure levels and dietary intake.

## Shaping the future

Projections based on the current trends, incidence velocity, risk-factor prevalence, population-attributable risks, and relative risk for risk factors concluded that by 2030, stroke will be the second leading cause of death globally, the first leading cause of death in middle-income countries and the third in low-income countries. 91

The stroke quadrangle is hereby proposed as a holistic synergy of four pillars aimed at reversing the rising burden. This approach is consistent with the successful high-impact interventions implemented in the United States over the past five decades,^68^ as well as the global stroke burden-reduction objectives from the World Stroke Association (http://www.worldstroke.org/) and the World Hypertension League (http://www.worldhypertensionleague.org/), which has resulted in a decline in stroke burden in high-income countries.^2^ It is expected that if resources are applied efficiently in a similar manner in LMICs, the burden of stroke will be reduced. These resources include:

• synergistic epidemiological surveillance and research networks exploring and monitoring trends in the burden, pattern and determinants (gene, environment, gene–gene, gene–environment, transcriptomics, etc) such as the Stroke Investigative Research and Educational Network (SIREN) project
• primordial, primary and secondary prevention programmes at individual, family, systems and community levels, e.g. the Tailored Hospital-based Risk reduction to Impede Vascular Events after Stroke (THRIVES) project,^10^,^92^,^93^ improvement of stroke literacy and early recognition
• acute stroke care facilities with rapid evacuation services
• stroke rehabilitation and recovery services.

## Conclusions

In contrast to the declining stroke rates in several developed countries, the incidence of stroke in Africa, especially haemorrhagic stroke, has risen substantially over the last 20 years. This rise can only be expected to continue unabated unless widespread coordinated efforts based on plausible paradigms that incorporate established and accumulating scientific evidence are promptly instituted.

The results of this assessment suggest intervention models such as ‘the stroke quadrangle’ implemented through the SIREN project may be an effective effort to catalyse risk reduction in this global high-risk population. SIREN is poised to identify the unique risk factors (genetic and environmental) associated with stroke occurrence, type, subtype, pattern and outcome in black Africans (in Africa and the USA). SIREN is designed to substantially enhance our understanding of factors that could be addressed to improve stroke outcomes, and possibly other vascular disease entities such as coronary artery disease and chronic kidney disease in people of African ancestry.

Over 3 000 case–control African pairs will be compared to 1 000 African-Americans and 12 000 white Americans in the Reasons for Geographic and Racial Differences in Stroke (REGARDS) study. The study aims to discover/explore potentially modifiable genetic pathways to stroke risk that may be common to people of African ancestry.

## Key messages

• Accurate epidemiological data on stroke in Africa is scanty.
• However, age-adjusted standardised annual stroke incidence rates may be up to 316 per 100 000, and age-adjusted standardised prevalence rates may be up to 981 per 100 000.
• From the Global Burden of Disease model-based estimates, stroke incidence appears to be increasing in Africa.
• Rigorous comprehensive and prospective epidemiological surveillance is urgently needed to assess and monitor the actual burden and determinants as well as the epidemiological trend of stroke in Africa.
• Appropriate intervention paradigms such as the stroke quadrangle are urgently required.
